# How to do it: investigate exertional rhabdomyolysis (or not)

**DOI:** 10.1136/practneurol-2018-002008

**Published:** 2018-10-10

**Authors:** Peter M Fernandes, Richard J Davenport

**Affiliations:** 1 Centre for Clinical Brain Sciences, University of Edinburgh, Edinburgh, UK; 2 Division of Clinical Neurosciences, University of Edinburgh, Western General Hospital, Edinburgh, UK

**Keywords:** myopathy, McArdle’s disease, metabolic disease, muscle disease, phosphofructokinase

## Abstract

Rhabdomyolysis is the combination of symptoms (myalgia, weakness and muscle swelling) and a substantial rise in serum creatine kinase (CK) >50 000 IU/L; there are many causes, but here we specifically address exertional rhabdomyolysis. The consequences of this condition can be severe, including acute kidney injury and requirement for higher level care with organ support. Most patients have ‘physiological’ exertional rhabdomyolysis with no underlying disease; they do not need investigation and should be advised to return to normal activities in a graded fashion. Rarely, exertional rhabdomyolysis may be the initial presentation of underlying muscle disease, and we review how to identify this much smaller group of patients, who do require investigation.

## Case 1

A 25-year-old male personal trainer ran a ‘fun run’ on a hot day wearing a heavy costume. He collapsed 9 km into the run and was brought to hospital. He was agitated and confused (Glasgow Coma Scale score 10; E3, V3, M4), feverish (40.5°C) and tachycardic (149 bpm) with muscle pain and weakness. He was cooled and given intravenous fluids. Investigations showed serum creatine kinase (CK) concentration was 3000 IU/L, rising to 105 000 IU/L at 24 hours, with an acute kidney injury and myoglobinuria. He was discharged 3 days later with no neurological deficit and normal renal function; his CK normalised after 3 weeks. He was an athletic man who had completed several marathons, with no medical conditions or family history of neuromuscular disease.

## Case 2

A 42-year-old male security supervisor attended a training course involving intense physical exertion. He became dehydrated and took oral and topical ibuprofen. He attended the emergency department 3 days afterwards with flank pain and dark urine. Neurological examination was normal but his serum CK was >300 000 IU/L and he required dialysis for acute kidney injury with myoglobinuria. He was discharged after 1 week with improving kidney function and CK 2750 IU/L. Repeat blood tests after 3 months showed normal kidney function and CK 334 IU/L, remaining elevated at 550 IU/L at 1 year. He had developed exercise intolerance in his teenage years without seeking medical attention. Further inquiries revealed his sister, with whom he had little contact for many years, had been diagnosed with McArdle’s disease in childhood. Subsequent gene testing demonstrated he was homozygous for the c.148C>T (p.Arg50Ter) pathogenic mutation in myophosphorylase (*PGYM*).

## Defining rhabdomyolysis

There is no universally agreed clinical definition of rhabdomyolysis, but a working definition would encompass the twin features of symptomatic muscle involvement and a substantial rise in serum CK, usually >50 000 IU/L. Muscle symptoms include myalgia, weakness and/or swelling. The presence of myoglobinuria is not required and many laboratories do not routinely measure serum and urinary myoglobin. Nevertheless, patients should be asked about changes in urine coloration, which may be the classical ‘Coca-Cola’ of myoglobinuria. Urine should be dipstick tested: haemoglobin without erythrocytes indicates myoglobinuria.

## Causes of rhabdomyolysis

Rhabdomyolysis can be divided into non-exertional and exertional causes (box 1), although certain non-exertional causes are due to generalised muscle overactivity, such as alcohol withdrawal, status epilepticus or tetany. Some causes, such as statin medications, may be obvious but others need more careful thought, as in case 2. A detailed history of the preceding activity is required, particularly for certain metabolic myopathies where events can be triggered by non-exertional muscle contraction, including emotional situations.^1^

Box 1 **Causes of rhabdomyolysis**

**Non-exertional causes**

- Drug
  - Amphetamines, cocaine, cyclosporine, fibrates, isoniazid, lithium, neuromuscular blocking agents, propofol, quetiapine, statins, zidovudine.
- Toxic
  - Alcohol, heavy metal poisoning, snake venom.
- Metabolic
  - Alcohol withdrawal, electrolyte abnormalities, hypothyroidism, serotonin syndrome, status epilepticus.
- Inflammatory
  - Dermatomyositis, polymyositis.
- Infection
  - Coxsackie, Epstein-Barr virus, influenza, HIV, malaria, tetanus, other viruses.
- Local muscle damage
  - Crush injury, compartment syndrome, muscle ischaemia.

**Exertional causes**

- Heat-related injuries (table 1, case 1)
  - Heat stroke, heat injury.
- Metabolic myopathies
  - Glycogenolytic disorders.
  - Fatty acid metabolism disorders.
  - Mitochondrial disorders.
  - Structural myopathies (eg, dystrophinopathies).

**Table 1:** Exercise-associated heat illnesses

Heat cramps	Exercise-associated muscle cramps are a mild form of exertional heat illness familiar to those who watch sports (except perhaps for darts aficionados) characterised by severe muscle pain and spasms/prolonged muscle contraction without other features.
Heat syncope	Exercise-associated transient loss of consciousness usually occurs after exercise cessation. The likely cause is a sudden reduction in venous return secondary to reduced skeletal muscle tone, and diversion of blood flow to extremities to lose heat. Recovery is rapid and complete (as in vasovagal syncope) and core body temperature is not elevated.
Heat exhaustion	Characterised by difficulty continuing with exercise with raised core body temperature but no significant or prolonged alteration of mental state.
Heat stroke	Combination of elevated core temperatures (above 40°C) and altered mental state. Other end organs may also be damaged, including muscles (raised serum CK), kidneys (acute kidney injury) and liver (elevated liver enzymes).
Heat injury	A description used by military physicians (but not recognised in ICD-10) to describe heat exhaustion and end-organ damage without mental state changes.

CK, creatine kinase; ICD-10, International Classification of Diseases, 10th Revision.

## Exercise-induced rhabdomyolysis

Exertional rhabdomyolysis is the combination of muscle symptoms (myalgia, weakness and swelling) and a substantial rise in serum CK (>50 000 IU/L) in the setting of exercise. Serum CK rises after exercise are common and are asymptomatic in up to half of cases.^2^ Mean serum CK values 24 hours after marathons are 3322 IU/L for men (22× baseline) and 946 IU/L for women (9× baseline).^3^ The clinical significance of the serum CK concentration is unclear: 6000 IU/L has been cited as the minimum necessary for renal failure^4^ but case reports indicate acute kidney injury may occur with a serum CK of 5000 IU/L^5^ and other studies suggest no safe serum concentration of CK.^6^ Pragmatically, a rise in CK to >5000 U/L and/or evidence of end-organ damage (eg, myoglobinuria or decline in renal/liver function) is sufficient for a diagnosis of exertional rhabdomyolysis. Patients with exertional rhabdomyolysis may also experience fever, nausea and decreased/absent urine production. Supportive biochemical findings include hyperkalaemia, hyponatraemia, hyperphosphataemia, hypercalcaemia or hypocalcaemia and metabolic acidosis.

Most cases of exertional rhabdomyolysis are caused by heat-related injuries, specifically heat stroke and heat injury. The WHO’s International Classification of Diseases, 10th Revision includes four overlapping categories relating to exertional heat conditions (table 1). These symptoms are worth enquiring about since rhabdomyolysis in the context of a clear-cut heat-related condition usually does not require further investigation.

## Consequences of exertional rhabdomyolysis

Exertional rhabdomyolysis causes traumatic and metabolic damage to myocytes, resulting in local muscle injury and systemic effects from release of intracellular contents. Acute kidney injury from myoglobin toxicity is a feared complication, but other serious consequences include compartment syndrome, hyperkalaemia and disseminated intravascular coagulation. Patients with preceding heat exposure may experience altered mental state, probably hyperthermia induced, which may be ameliorated by adequate hydration.^7^ The consequences of untreated exertional rhabdomyolysis are described in the earliest account of this condition, from the Roman invasion of southern Arabia in 24 BC led by Aelius Gallus:

The desert, the sun, and the water … caused his men great distress, so that the larger part of the army perished. The malady … attacked the head and caused it to become parched, killing forthwith most of those who were attacked, but in the case of those who survived this stage it descended to the legs, skipping all the intervening parts of the body, and caused dire injury to them.

Dio Cassus (150–235 AD)^8^

Rates of renal failure after exertional rhabdomyolysis vary between studies, in keeping with uncertain diagnostic criteria for both acute kidney injury and exertional rhabdomyolysis. A study of 1203 US Army soldiers with exertional rhabdomyolysis found that 8% developed renal failure^9^; a civilian case series of 475 patients with rhabdomyolysis reported 46% had acute kidney injury.^10^ A Danish study of 161 rhabdomyolysis cases found 27% were exercise induced, mostly from weight training rather than endurance exercise; none had significant acute kidney injury.^11^ There appears to be a rising incidence of exertional rhabdomyolysis, perhaps related to societal changes in exercise preference^12^; ‘rhabdo’ is now well recognised among exercise enthusiasts and articles have appeared in the lay press in recent years with the advent of high-intensity workouts such as Spin classes and CrossFit.^13^ The outcome in these cases is good, with little evidence of permanent renal dysfunction.^6^

The psychological impact of a major health event on an otherwise well person should not be neglected. The patient in case 1 found the experience sufficiently traumatic that he could not exercise, having to change career.

## Incidence and risk factors for exertional rhabdomyolysis

It is challenging to define the incidence of exertional rhabdomyolysis since many patients do not present to medical attention. The US Army study suggested an annual incidence of 7/10 000, although in a highly selected group.^9^ Risk factors for greater rises in serum CK after exercise include low premorbid physical fitness, male sex, African ethnicity, dehydration and high-intensity prolonged weight-bearing exercises, particularly eccentric muscle contractions (muscle contraction during muscle lengthening, eg, downhill running).^14^ Any impairment of heat loss increases the risk of exertional rhabdomyolysis, including environmental factors (higher ambient temperatures, clothing) and use of vasoconstrictive drugs (amphetamines). Non-steroidal anti-inflammatory medications do not increase the risk of exertional rhabdomyolysis but permit exercise to continue beyond normal limits and so increase the risk of acute kidney injury (case 2).

The risk of exertional rhabdomyolysis is increased up to 11-fold in those with previous heat injuries and over 50% of patients with exertional rhabdomyolysis have a history of heat cramps or heat exhaustion.^9^ Sickle-cell trait increases risk with HR 1.54 (95% CI 1.12 to 2.12)^15^; fatal exertional rhabdomyolysis has also occurred in athletes with sickle-cell trait.^16^ Other risk factors include obesity (HR 1.39, 95% CI 1.04 to 1.86), tobacco smoking (HR 1.54, 95% CI 1.23 to 1.94), antipsychotic medications (HR 3.02, 95% CI 1.34 to 6.82) and statins (HR 2.89, 95% CI 1.51 to 5.55).^15^ The colloquial farming term of ‘Monday morning disease’ derives from the higher risk of exertional rhabdomyolysis after prolonged rest or carbohydrate-rich diets in horses, though this is likely secondary to an underlying metabolic myopathy.^17^

## Acute treatment of exertional rhabdomyolysis

The management of exertional rhabdomyolysis depends on the clinical presentation, with extensive coverage of treatment options beyond the scope of this article. Clinicians should have a low threshold for referral to the intensive care unit, particularly where the serum CK concentration is >10 000 U/L, the patient needs active cooling, or there is end-organ dysfunction such as obtundation.^18^ Giving high-volume intravenous fluids early, aiming for urine outputs of above 200 mL/hour, seems beneficial^19^; serum electrolytes should be checked frequently. Urinary alkalinisation improves myoglobin removal, though there is no evidence that sodium bicarbonate or mannitol helps.^5^ Plasma exchange—but not haemodialysis— removes myoglobin. Compartment syndrome requires immediate referral to orthopaedics or plastic surgery. All potentially causative drugs should be stopped, including statins, non-steroidal anti-inflammatory drugs, selective serotonin reuptake inhibitors, supplements (weight loss treatments or creatinine), recreational drugs and others.^20^

After discharge, the serum CK and renal function should be monitored every 72 hours; the CK or creatinine may not peak until after day 4.^14^ Monitoring can stop when the serum CK returns to below 1000 IU/L, though its failure to return to normal concentration should prompt consideration of underlying myopathy, as in case 2.

## Investigating exertional rhabdomyolysis

In general, patients with a clear history of a preceding heat-related illness do not require further investigations; this group is statistically the largest. Further investigations should be considered in patients with no history of heat exposure, or who have recurrent exertional rhabdomyolysis. Patients may not have sought medical attention for previous milder episodes; ask about previous episodes of postexertional muscle pain, weakness or dark urine. Almost all patients with metabolic myopathies will have had symptoms since teenage years. Other features requiring further investigation include persistently raised serum CK after 8 weeks, family history of rhabdomyolysis (including after exercise, fasting or illness), exertional muscle cramps or exercise intolerance. The acronym (retronym?) RHABDO has been suggested as an aide-mémoire (box 2).^18^

Box 2 **Features suggesting need for further investigation^18^**

R—Recurrent episodes of exertional rhabdomyolysis

H—HyperCKaemia more than 8 weeks after event

A—Accustomed to exercise

B—Blood creatine kinase (CK) concentration above 50× upper limit of normal

D—Drug ingestion insufficient to explain exertional rhabdomyolysis

O—Other family members affected or Other exertional symptoms

**Figure 1:**
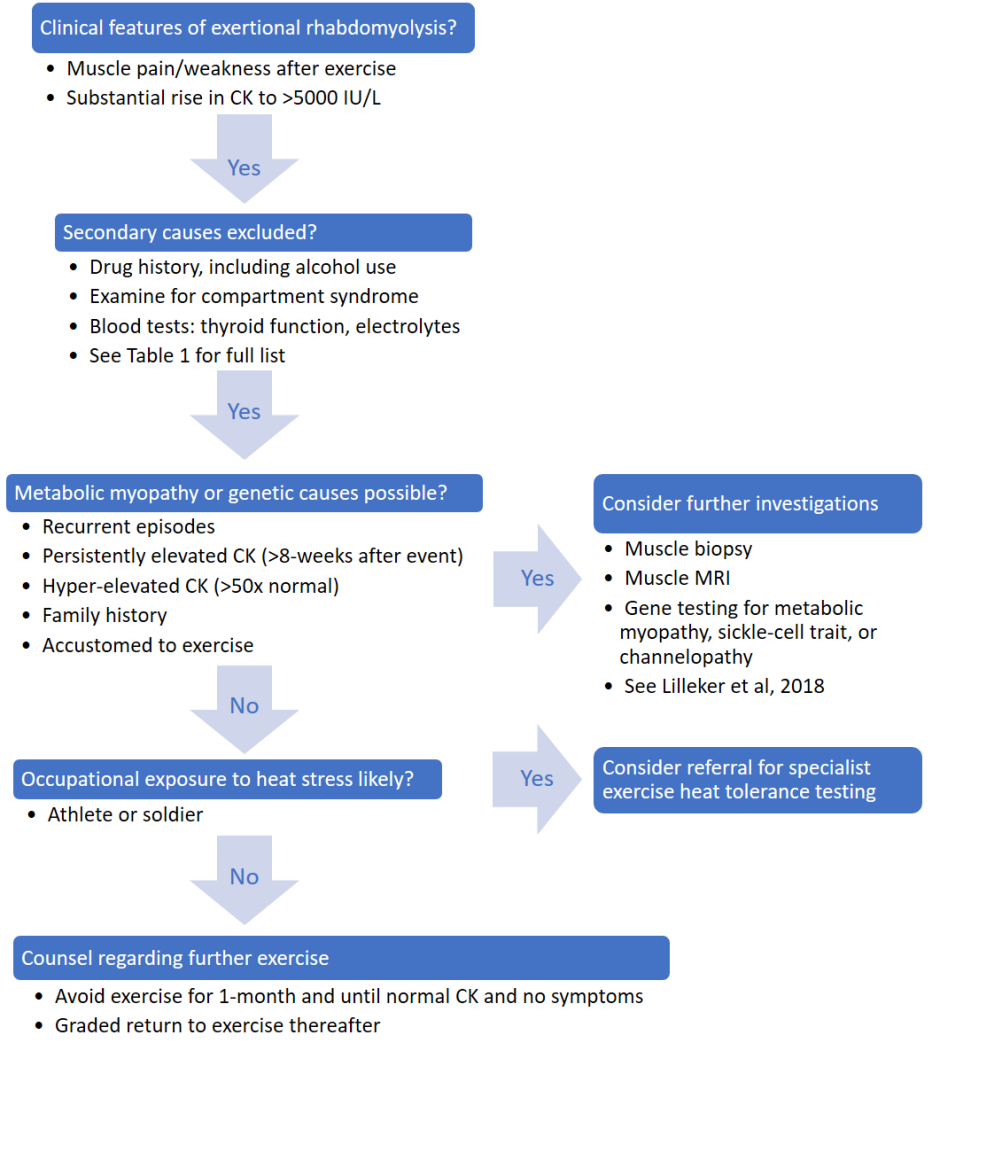
A suggested approach to investigating exertional rhabdomyolysis. CK, creatine kinase.

Clinicians should take a general myopathy history, including exercise intolerance, exertional rhabdomyolysis tempo (eg, duration between exercise and onset) and family history. Family history is sometimes forgotten: case 2 had little contact with his sister but recalled she had been diagnosed with something which stopped her exercising; renewed contact revealed her McArdle’s disease. Examination for myopathic weakness and electromyography should be delayed until the patient has fully recovered. Further investigations for underlying causes, including exercise testing, muscle biopsy and muscle MRI, depend on the clinical phenotype but are often non-diagnostic until the exertional rhabdomyolysis has resolved. Figure 1 summarises the approach to a new presentation of exertional rhabdomyolysis.

A full discussion of metabolic myopathies causing rhabdomyolysis is beyond the scope of this article, but has been covered elsewhere^21–23^ and in table 2. Structural myopathies, including dystrophinopathies, limb-girdle muscular dystrophy 2I and dysferlinopathies, can present with exertional rhabdomyolysis.^22^ Many metabolic myopathies are diagnosed relatively late in life, with the Spanish McArdle’s disease cohort having an average age of 44 years at diagnosis.^24^ This is usually due to a delay in presentation or diagnosis rather than a lack of symptoms: as with most metabolic myopathies, most patients (86%) develop symptoms in childhood (usually exercise intolerance). Patients with symptom onset in later life are unlikely to have a metabolic myopathy, though subtle previous events may only be revealed with careful history taking.

**Table 2:** Summary of inherited metabolic causes of exertional rhabdomyolysis

Metabolic pathway	Trigger	Baseline CK	Baseline weakness	Examples
Glycogen metabolism	Early onset after intense exercise	Often high	May develop in later life	McArdle’s disease (second wind phenomenon) Tarui’s syndrome (↑exercise tolerance when fasting, compensated haemolysis) Lactate dehydrogenase-A deficiency (photosensitive rash)
Fatty acid metabolism	Later onset after prolonged exercise or illness/fasting	Normal	Unusual	Carnitine palmitoyltransferase (diffuse exertional weakness, respiratory failure)
Mitochondrial metabolism	Early or late onset, prominent fatigue	Normal or high	Possible	Coenzyme Q10 (encephalopathy, ataxia, convulsions) Cytochrome B/C (onset after mild exercise)

CK, creatine kinase.

Channelopathies, including *RYR1* gene mutations associated with malignant hyperthermia, may cause exertional rhabdomyolysis.^25^ Rhabdomyolysis in patients with the *RYR1* gene may be triggered by heat exposure without exercise, illness and alcohol. Identifying these patients is important because of the risks associated with administering certain anaesthetic agents. Other gene mutations associated with exertional rhabdomyolysis include *ACE*, *ACTN3*, *CCL2* and *CCR2*.^18^ Patients with some of these mutations may have supranormal athletic abilities, indicating a trade-off between enhanced exercise aptitude and risk of exertional rhabdomyolysis. The presence of sickle-cell trait should be investigated in those with an appropriate ethnic background; the UK newborn screening programme should detect most cases.

## Gene panel

The Sheffield (UK) centre offers a rhabdomyolysis and metabolic myopathy 30-gene panel. The standard test takes 16 weeks at an NHS cost of £900^26^ and sequences the entire coding regions of 30 genes, including *PYGM* (McArdle’s disease), *PFK* (Tarui’s disease) and other glycogenolytic, fatty acid and mitochondrial metabolism genes; structural myopathies and channelopathies are not included.

## Role of exercise heat tolerance tests

Heat tolerance testing is usually restricted to military or professional athletic circles. Protocols differ but consist of prolonged static exercise (treadmill or bicycle) in a controlled environment with invasive temperature monitoring. A ‘failed’ or positive test is where an increase in temperature occurs earlier, at a faster rate, and to a greater degree (>38.6°C), than the normal population, accompanied by tachycardia (>160 bpm). There is little evidence on the usefulness of these tests, but studies by the Israeli Defence Forces indicate a minimum duration of 120 min under hot (40°C) conditions.^27^ While a useful screening test in the right circumstances, exercise heat tolerance tests are likely to be replaced by gene panel testing in many situations.

## Returning to normal activities

The risk of recurrence is low if there is no suggestion of an underlying genetic cause: 1%–2% suffered recurrent exertional rhabdomyolysis in the US Army study,^9^ the risk may be lower in the general population.

Most experts recommend a graded return to exercise.^18 28^ Exercise should be avoided in the first month and until symptoms have disappeared and the serum CK normalised. Light exercise can then be started and gradually increased in extent and duration if symptoms of weakness and/or myalgia do not recur. Eccentric training is best avoided, at least to start with, as is strenuous unaccustomed exercise. Patients with suspected underlying myopathies/genetic disorders need a stricter approach, as described elsewhere.^18^

## Further reading

- *Diagnostic evaluation of rhabdomyolysis*. Nance JR, Mammen AL. *Muscle & Nerve* (2015) 51:793-810
- *Exertional rhabdomyolysis: physiological response or manifestation of an underlying myopathy?* Scalco RS, Snoeck M, Quinlivan R, Treves S, Laforet P, Jungbluth H, Voermans NC. *BMJ Open Sport Exerc Med* (2016) 2:e000151
- *Metabolic myopathies: a practical approach.* Lilleker JB, Keh YS, Roncaroli F, Sharma R, Roberts M. *Pract Neurol* (2018) 18:14-21

**Key points**

- Exertional rhabdomyolysis is potentially life threatening.
- Risk factors include unaccustomed exercise, eccentric exercise, sickle-cell trait, underlying genetic disorders and previous heat-related illnesses.
- Treatment involves aggressive fluid resuscitation and may necessitate intensive care.
- Patients with recurrent events, elevated baseline serum creatine kinase, or features consistent with metabolic myopathies should be considered for investigation for underlying genetic disorders.
- Rhabdomyolysis in the setting of a heat syndrome usually does not require further muscle investigations.
- Patients should return to normal activities in a graded fashion.
